# Editorial: Renewed insight into cancer mechanism and therapy

**DOI:** 10.3389/fonc.2025.1710803

**Published:** 2025-10-10

**Authors:** Yihao Liu, Lu Pan, Li Huang, Qinqin He, Hongbing Zhang, Jiadi Gan

**Affiliations:** ^1^ Department of Biotherapy, Cancer Center, West China Hospital, Sichuan University, Chengdu, China; ^2^ Department of Lung Cancer Surgery, Tianjin Medical University General Hospital, Tianjin, China; ^3^ Department of Pain Management, West China Hospital, Sichuan University, Chengdu, China; ^4^ Department of Respiratory and Critical Care Medicine, West China Hospital, Sichuan University, Chengdu, China

**Keywords:** immunotherapy, targeted therapy, personalized treatment, tumor resistance, clinical translation

## 1 Introduction

Advances in the fields of molecular biology, immunology, and genomics have driven substantial improvements in cancer therapy, especially in our comprehension of oncogenic processes and the discovery of new therapeutic targets. These scientific developments have reinvigorated the field, with notable progress in the implementation of targeted therapies, immune-based treatments, and patient-specific strategies. Nonetheless, the long-term effectiveness of such approaches is often undermined by tumor relapse and the emergence of drug resistance. As a result, understanding how cancers evade immune destruction, identifying alternative molecular targets, and refining personalized treatment protocols remain central challenges in the current oncology landscape.

Our research theme “Renewed Insight into Cancer Mechanism and Therapy” was conducted in Frontiers in Oncology, with the aim of promoting the publication of more research on cancer therapy methods and the mechanisms of tumorigenesis and development. The theme has now concluded, and this editorial provides a summary of the research under this theme.

## 2 Immune evasion and therapeutic counterstrategies

The immune system plays an essential role in identifying and eradicating malignant and abnormal cells via highly specialized mechanisms. The well-established “3E” model—comprising elimination, equilibrium, and escape—captures the dynamic interaction between cancer cells and immune surveillance (1). Initially, immune cells such as macrophages and natural killer (NK) cells eliminate cancerous cells through cytotoxic processes involving enzymes like perforin and granzymes. T lymphocytes, particularly CD8^+^ cytotoxic T cells and CD4^+^ helper subsets, are critical players in adaptive immunity. Additionally, B cells contribute by generating antibodies that mediate antibody-dependent cellular cytotoxicity (ADCC). However, some tumors manage to circumvent this immune clearance, advancing into equilibrium or escape phases—often due to impaired antigen presentation or lack of specific immune cell populations, which may be influenced by the host’s immune status (2).

A newer framework, referred to as the “3C” model—Camouflage, Coercion, and Cytoprotection—has been suggested to better explain immune evasion tactics (3). Tumors may initially escape detection by altering or shedding antigens. Once this fails, they can suppress immune activity by manipulating immunosuppressive signaling or reprogramming local metabolism. Furthermore, tumor cells may protect themselves via mechanical means such as disrupting immune synapses or by disabling apoptotic pathways to ensure continued growth and spread.

Adoptive cell therapy (ACT) has shown promise in overcoming such immune evasion (4). This therapeutic strategy encompasses tumor-infiltrating lymphocytes (TILs), chimeric antigen receptor T (CAR-T) cells, and T-cell receptor-modified (TCR-T) cells, all designed to boost immune specificity and killing capacity. Ruan et al. have emphasized the therapeutic value of ACT by modifying autologous or donor-derived immune cells. Additionally, research by Cai et al. indicates that hypermethylation-induced silencing of the STING pathway can prevent immune infiltration into tumors, suggesting that demethylating drugs may be viable tools to reinvigorate anti-tumor immunity and combat resistance.

## 3 Future directions in targeted and innovative oncology therapies

Progress in molecular oncology has significantly expanded our understanding of tumor-specific gene alterations and protein-level abnormalities. Distinct mutational profiles across different malignancies justify the ongoing development of targeted drugs. Lung cancer remains the most lethal cancer worldwide (5). In Asian populations, epidermal growth factor receptor (EGFR) mutations are particularly prevalent among NSCLC patients (6). Osimertinib, a third-generation EGFR tyrosine kinase inhibitor (TKI), is effective against resistance mutations and shows enhanced central nervous system activity (7). In resectable NSCLC, it has been shown to improve both disease-free survival (DFS) and overall survival (OS) when compared to standard chemotherapy. However, findings from a phase II trial assessing neoadjuvant osimertinib in stage I–IIIA patients were suboptimal, indicating the potential benefit of combining this agent with cytotoxic chemotherapy. KRAS mutations are also common drivers in NSCLC (8). Agents like sotorasib and adagrasib have yielded promising results, although their use with immune checkpoint inhibitors has raised safety concerns, particularly hepatotoxicity. These drugs nevertheless remain potential options for KRAS-driven disease. A smaller subset of NSCLC patients presents with ALK rearrangements, which are linked to high rates of brain metastasis (7). ALK-targeting agents such as crizotinib and alectinib have demonstrated significant efficacy in delaying CNS involvement and improving clinical outcomes. Furthermore, targeted therapies directed at ROS1 fusions, NTRK gene rearrangements, MET exon 14 skipping mutations, and HER2 alterations continue to broaden the therapeutic landscape, particularly with next-generation TKIs developed to address resistance (6).

In advanced gastric cancer, the standard front-line approach remains platinum- and fluoropyrimidine-based chemotherapy (9). However, molecular classification has allowed the integration of targeted agents (10). HER2-positive tumors respond well to trastuzumab-based regimens, and newer drugs such as trastuzumab deruxtecan offer superior antitumor effects (11, 12). Anti-angiogenic therapies targeting VEGF/VEGFR—such as bevacizumab and sorafenib—aim to inhibit tumor vasculature and have been utilized across multiple cancer types. FGFR2b, an isoform involved in embryogenesis and tissue repair, is abnormally expressed in several cancers. Bemarituzumab has demonstrated OS benefits nearing 30 months in HER2-negative gastric tumors. Additionally, MET-amplified gastric cancer patients have shown favorable responses to savolitinib monotherapy.

Hepatocellular carcinoma (HCC), a leading digestive malignancy, is most commonly treated with the multikinase inhibitor sorafenib (13). However, resistance remains a formidable challenge. Current efforts are focused on combining TKIs with immunotherapy or chemotherapy to improve survival metrics while minimizing adverse events.

Intriguingly, Fang et al. highlighted the influence of the tumor-resident microbiome on cancer progression and therapeutic response. While some microbial species are oncogenic, engineered bacteria could potentially enhance anti-cancer immunity, though clinical safety remains a concern. Miao et al. discussed inflammation-related cardiac adverse events post-treatment and advocated for inflammation-based markers to monitor toxicity severity.

## 4 Personalized approaches in oncology

The evolution of precision medicine has redirected cancer treatment toward highly tailored, molecularly guided interventions. Immunotherapy and targeted approaches are now central to therapeutic decision-making based on individual genetic alterations. Novel agents aimed at immune checkpoints, RET fusions, BRAF mutations, and NTRK rearrangements are continually being refined to increase efficacy and reduce resistance. Case reports continue to demonstrate the practical potential of precision oncology. Liu et al. described a patient with malignant phyllodes tumor (MPT) and pleural spread treated with tislelizumab, achieving progression-free survival of 3.5 months and an overall survival of 11.1 months. Likewise, Shen et al. reported a MET-amplified metastatic gastric cancer patient who initially responded well to crizotinib before experiencing relapse due to acquired resistance. Although long-term control was limited, these examples highlight the relevance of personalized treatment even in aggressive, advanced disease. Multidisciplinary collaboration may further improve therapeutic outcomes and reduce treatment-related morbidity.

## 5 Conclusion

Multiple therapeutic strategies are currently available for managing malignancies. The continued identification of novel biomarkers and targetable mutations is pivotal for advancing cancer care. Rational combinations of chemotherapy, immunotherapy, and molecular targeting can improve response rates, though they may also increase adverse events. Consequently, devising optimal, patient-specific regimens remains an ongoing clinical challenge. A deeper understanding of the mechanisms underlying resistance to immunotherapies and targeted drugs will be essential to overcoming immune escape. While personalized therapy may not be universally applicable, it holds considerable promise, particularly when guided by genomic profiling, in delivering more effective and durable responses in select patient subsets.
